# Parents’ perception of auditory hypersensitivity in children with Autism Spectrum Disorder

**DOI:** 10.1192/j.eurpsy.2025.1100

**Published:** 2025-08-26

**Authors:** C. J. S. Ribeiro, L. D. Bezerra, C. A. D. L. H. Amato

**Affiliations:** 1 Universidade Presbiteriana Mackenzie, São Paulo, Brazil

## Abstract

**Introduction:**

Autism is defined as a broad and complex neurodevelopmental disorder with alterations in behavioral and social communication aspects. Sensory symptoms also occur prevalently in autism spectrum disorder (ASD) and are present early in the etiology, however, little is known about the early developmental patterns of these symptoms (Mccormick *et al*. Autism 2016; 5 572-579). Among these various sensory-perceptual alterations, auditory hypersensitivity is a prevalent sensory alteration in the ASD population (Dunlop *et al*. Front Hum Neurosci 2016; 10 1-12), defined as excessive or abnormal sensitivity and distress to auditory stimuli that are evident in the individual’s behavioral reactions (Stefanelli *et al*. CoDAS 2020; 32 1-9). Furthermore, auditory hypersensitivity can trigger atypical reactions that can impact social and academic domains (Danesh *et al*. *Audiol. Res 2021; 11* 547-556).

**Objectives:**

This research aimed to identify the occurrence and describe the auditory hypersensitivity behaviors presented by children with Autism Spectrum Disorder.

**Methods:**

This is a cross-sectional and descriptive study, composed of parents/guardians of 161 children diagnosed with ASD. The sample consisted of parents of children of both sexes, aged 4 to 12 years. Caregivers answered a sociodemographic form and reported on the presence or absence of auditory hypersensitivity and the behaviors presented by the child. The study was approved by the Ethics and Research Committee under opinion number: 5,862,943.

**Results:**

161 parents of children with ASD participated, who declared that 108 (67.1%) of the children had auditory hypersensitivity and 53 (32.9%) did not, with 83 (69.2%) of the children being male and 37 (30.8%) female. The most frequently reported behaviors were: difficulty sleeping (79.8%), difficulty concentrating (76.8%), put your hands over ears (74.8%), nervousness (74.6%), fatigue and stress (72%), and irritability (71.8%) in the presence of sound (Table 1).

Tabela 1. Comportamentos de hipersensibilidade auditiva na percepção de pais de crianças com TEA

**Image 1:**

**Figure:**
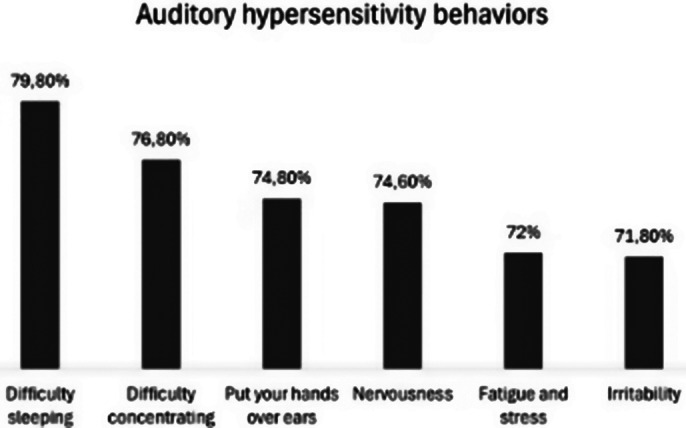

**Conclusions:**

The results suggest that there is a high prevalence of auditory hypersensitivity in children with autism spectrum disorder and that this symptom can have negative impacts on the individual’s quality of life.

**Disclosure of Interest:**

None Declared

